# Indoles Derived From Glucobrassicin: Cancer Chemoprevention by Indole-3-Carbinol and 3,3'-Diindolylmethane

**DOI:** 10.3389/fnut.2021.734334

**Published:** 2021-10-01

**Authors:** David E. Williams

**Affiliations:** Department of Environmental and Molecular Toxicology, Linus Pauling Institute, Oregon State University, Corvallis, OR, United States

**Keywords:** glucobrassicin, indole-3-carbinol, cancer, chemoprevention, pharmacokinetics, 3, 3'-diindolylmethane

## Abstract

Hydrolysis of glucobrassicin by plant or bacterial myrosinase produces multiple indoles predominantly indole-3-carbinol (I3C). I3C and its major *in vivo* product, 3,3'-diindolylmethane (DIM), are effective cancer chemopreventive agents in pre-clinical models and show promise in clinical trials. The pharmacokinetics/pharmacodynamics of DIM have been studied in both rodents and humans and urinary DIM is a proposed biomarker of dietary intake of cruciferous vegetables. Recent clinical studies at Oregon State University show surprisingly robust metabolism of DIM *in vivo* with mono- and di-hydroxylation followed by conjugation with sulfate or glucuronic acid. DIM has multiple mechanisms of action, the most well-characterized is modulation of aryl hydrocarbon receptor (AHR) signaling. In rainbow trout dose-dependent cancer chemoprevention by dietary I3C is achieved when given prior to or concurrent with aflatoxin B_1_, polycyclic aromatic hydrocarbons, nitrosamines or direct acting carcinogens such as N-methyl-N'-nitro-nitrosoguanidine. Feeding pregnant mice I3C inhibits transplacental carcinogenesis. In humans much of the focus has been on chemoprevention of breast and prostate cancer. Alteration of cytochrome P450-dependent estrogen metabolism is hypothesized to be an important driver of DIM-dependent breast cancer prevention. The few studies done to date comparing glucobrassicin-rich crucifers such as Brussels sprouts with I3C/DIM supplements have shown the greater impact of the latter is due to dose. Daily ingestion of kg quantities of Brussels sprouts is required to produce *in vivo* levels of DIM achievable by supplementation. In clinical trials these supplement doses have elicited few if any adverse effects. Sulforaphane from glucoraphanin can act synergistically with glucobrassicin-derived DIM and this may lead to opportunities for combinatorial approaches (supplement and food-based) in the clinic.

## Introduction

**Figure 1:**
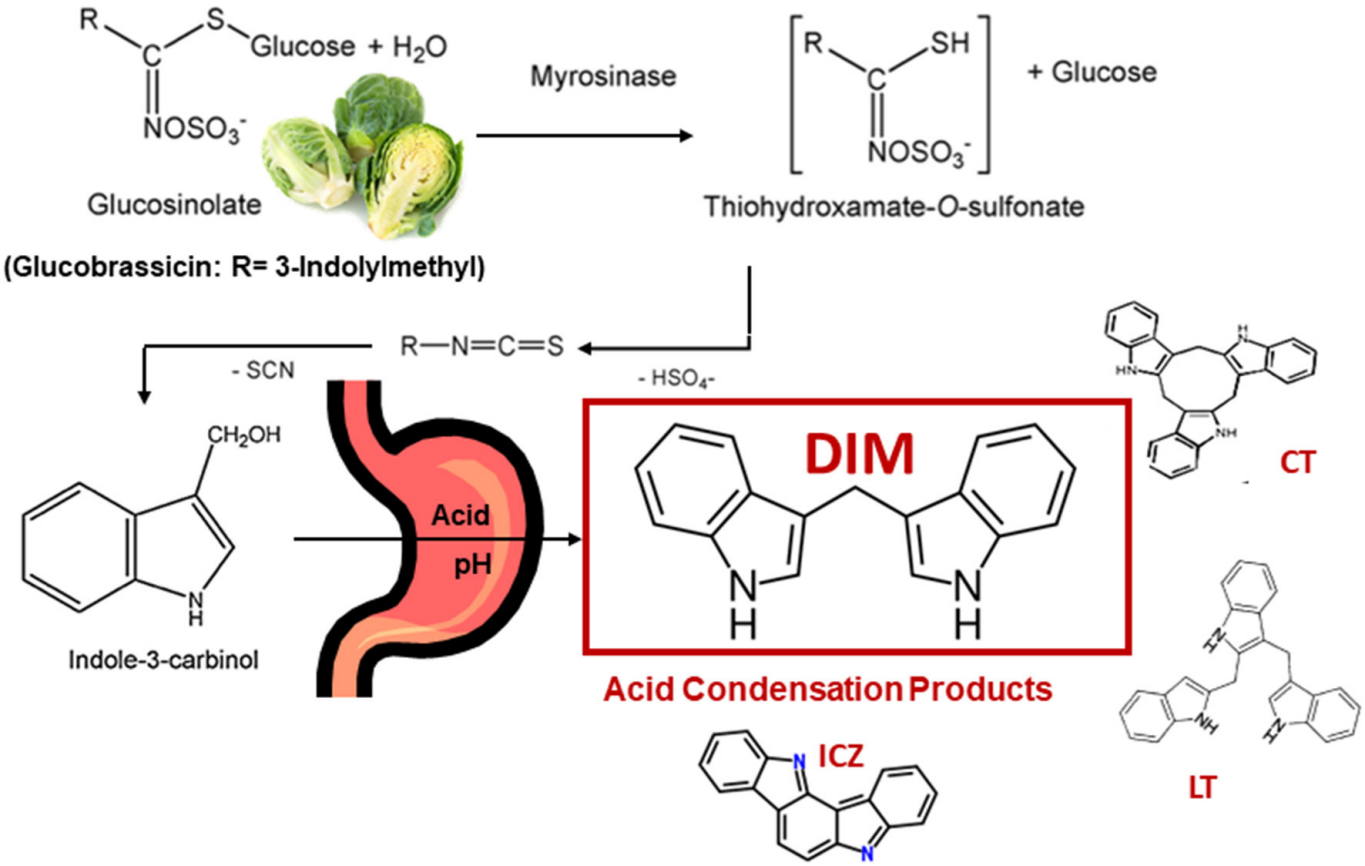
Cleavage of Glucobrassicin in Cruciferous Vegetable by Myrosinase, Formation of Indole-3-Carbinol (I3C) and Examples of Acid Condensation Products Produced *in vivo*. 3,3'-Diindolylmethane (DIM) is the Major Product. Cyclic CT) and Linear Trimers (LT) as well as Indolo[3,2-*b*]carbazole (ICZ) are Present in Trace Amounts.

Glucosinolates are found primarily in cruciferous vegetables (1–3). This review will focus primarily on the hydrolysis product of glucobrassicin, indole-3-carbinol (I3C), and the major *in vivo* I3C product, 3,3'-diindolylmethane (DIM). Glucobrassicin (Figure 1) is one of dozens of glucosinolates and is especially rich in the crucifer family. Brussels sprouts contain 1.6–2.7 mg/g dry weight glucobrassicin (4). Myrosinase (β-thioglucoside glucohydrolase, EC 3.2.3.1), present in plant cells (and in some gut bacteria), hydrolyzes glucobrassicin to the unstable indole-3-methylisothiocyanate which spontaneously decomposes yielding primarily indole-3-acetonitrile and I3C (Figure 1). On a molar basis the yield of I3C from glucobrassicin is about 20% corresponding to 0.11–0.18 mg/g dry weight for Brussels sprouts.

### Acid Condensation Products: Should I3C Be Considered a “Pronutraceutical”?

I3C is subject to decomposition especially under acidic conditions (5). In the stomach, I3C undergoes extensive condensation to yield a mixture of DIM as well as linear and cyclic trimers and tetramers (2, 3, 5, 6) (Figure 1). DIM predominates as the major product under acidic conditions both *in vitro* and *in vivo* (5, 6). The pharmacological activity of I3C is markedly reduced or eliminated if exposure bypasses the stomach strongly suggesting beneficial properties of I3C, such as cancer chemoprevention, are not due to I3C itself but rather component(s) of the acid condensation mixture (6). Unfortunately, with the exceptions of one linear trimer [LT, 2-(indol-3-ylmethyl)-3,3'-diindolylmethane], one cyclic trimer [CT, 5,6,11,12,17,18-hexahydrocyclononal (1,2-*b*:4,5-*b'*:7,8-*b”*) triindole] and indolo[3,2-*b*]carbazole (ICZ), the pharmacological activities of individual acid condensation mixture products have not been described to date. The focus of the rest of this review will be on the efficacy and mechanism(s) of action for I3C and DIM *in vitro* and *in vivo* as cancer chemoprevention agents. A number of excellent reviews on I3C/DIM in cancer chemoprevention, as well as potential therapeutic agents for other disease, can be found in the literature and those cited here are not a complete list (7–18). A comprehensive set of abstracts on DIM research can be found at the DIM Information Resource Center (www.diindolylmethane.org/references.htm).

### *In vitro* Studies

There have been numerous studies employing cancer cell lines of I3C and DIM potency and efficacy as well as mechanism(s) of action as anticarcinogens. Given that I3C spontaneously dimerizes to DIM even at neutral pH (19), there is the question of physiological relevance in studying I3C in cell-based systems and this is reinforced by the observation that I3C is rarely, if ever, detected in blood after oral ingestion. A reasonable presumption is that a portion, if not the majority, of the cellular effects reported following incubation with I3C, especially with long incubations at concentrations typically ≥100 μM, are due to DIM rather than I3C. The relevance of many *in vitro* studies of DIM (typically at concentrations of 10–100 μM) in cancer cells could also be questioned as plasma levels following ingestion of supplements have been reported to be ≤ 1 μM and recent evidence in humans show that a significant portion exists as DIM metabolites with unknown pharmacological activity (20).

I3C/DIM impacts cancer cells in numerous ways including inhibition of proliferation, stimulation of apoptosis and alteration of cell cycle control (9, 16–33). DIM is a weak agonist of the aryl hydrocarbon receptor (AHR) with K_d_ of 90 nM (34). ICZ, by comparison, is a strong agonist with a K_d_ of 0.19 nM (5). However, the concentrations of ICZ achievable *in vivo* are much lower than with DIM ( ≤ 1 μM). One cancer cell line that has been extensively studied with DIM is the MCF-7 human breast cancer cell line as, in addition to AHR, DIM impacts estrogen receptor (ER)-dependent signaling in these ER-regulated cells (35–45). Riby et al., demonstrated that the CT acid condensation product is an ER agonist (EC_50_ = 100 nM in MCF-7 cells (39). As AHR and ER exhibit “cross-talk” through a number of mechanisms, with a resultant impact on breast cancer cells (45–47), it is not surprising that human trials have examined chemoprevention of breast cancer (7, 8, 48). DIM is an antagonist of the androgen receptor which has also led to a focus on prostate as a target for I3C/DIM chemoprevention (45, 49). One target gene for DIM-dependent AHR activation is cytochrome P450 (CYP) 1B1 which is effective in estrogen hydroxylation at the 2- and 4-positions (50). The net result of DIM-dependent AHR modification of E_2_ metabolism is a decrease in the ratio of 16α/2-hydroxy-E_2_ and concomitant decrease in E_2_ levels (51). 16α-Hydroxy-E_2_ retains estrogenic activity and has been referred to as the “bad” E_2_ metabolite whereas 2-hydroxy-E_2_ has been termed the “good” E_2_ metabolite. The ratio of 16α/2-hydroxy-E_2_ and total E_2_ levels are often used as biomarkers for DIM in chemoprevention studies of estrogen-driven cancers such as breast (51, 52).

The large number of targets demonstrated for I3C and DIM in chemoprevention are likely due, in part, to epigenetic mechanisms in control of gene expression in addition to ligand binding to transcription factors such as AHR, ER, AR, Sp1, Nrf-2 (both directly and via AHR) and NFκB (33, 53, 54). Modulation of the expression and/or activities of components of epigenetics e.g., DNA methyl transferases (DNMTs), histone deacetylases (HDACs), and non-coding RNAs (miRNAs and lncRNAs) may be dependent or independent of I3C/DIM ligand transcription factor activation. Increased DNA methylation often silences tumor suppressor genes and amelioration of this silencing is thought to be an important mechanism in cancer chemoprevention. DIM was effective in down-regulation of Dnmt/DNMT in mouse and human prostate cancer cells (55, 56) and repressing DNMT in normal prostate (PrEC) as well as androgen-dependent (LnCAP) or androgen-independent (PC3) prostate cancer cells (56). DIM-dependent inhibition of DNMT tended to down-regulate expression of oncogenes while enhancing tumor suppressor genes (55). In mouse TRAMP C1 prostate cancer cells, DIM reduced expression of both mRNA and protein for Dnmt1, Dnmt3a and Dnmt3b (55).

DIM was found to selectively down-regulate protein levels of class I HDACs (HDAC1, HDAC2, HDAC3 and HDAC8) in human colon cancer and prostate cancer cell lines and the mechanism is enhanced proteosomal HDAC degradation (57, 58). Most HDAC inhibitors [e.g., suberoylanilide hydroxamic acid (SAHA) and trichostatin] are competitive enzyme activity inhibitors and this novel mechanism exhibited by DIM could be used in the clinic with HDAC enzyme inhibitors to more effectively reduce histone acetylation and/or lower the concentrations of HDAC inhibitors required. As with other HDAC inhibitors, DIM could enhance AHR-mediated gene transcription by a mechanism other than ligand binding. In both mouse colonocytes and human Caco-2 cells, HDAC inhibitors including butyrate, Panobinostat and Vorinostat (SAHA) synergistically enhanced ligand- (including indole) dependent AHR induction of CYP1A1 and other AHR target genes (59). HDAC and DNMT together contribute to the balance of permissive (H3K4me3) or repressive (H3K27me3) chromatin marks (60) and the bivalent nature of these marks is impacted *in vitro* by DIM.

I3C and DIM modulate expression of a number of miRNAs and lncRNAs (53, 54). The miRNAs regulated by DIM include let-7a-e, miRNA-15a, miRNA-16, miRNA17-5p, miRNA-19a, miRNA-20a, miR-21, miR-27b, miR-30e, miR-31, miR-34a, miRNA-92a, miRNA-106a, miR-124, miR-146, miRNA-181a, miRNA-181b, miRNA-210, miR-219, miRNA-221, miR-320, miR-490, miR-495 and miR-1192 (11, 53, 60–63). Not surprisingly, miRNAs known to be tumor suppressors tend to be up-regulated while oncomirs are down-regulated (64). A number of the target genes are important in regulation of the cell cycle and apoptosis. LncRNAs regulated by DIM include PCGEM1, HOTAIR and CCAT-L (65–67). This induction/repression of miRNAs and LncRNAs may be due, in part, to DIM-dependent binding to transcription factors such as AHR or AR. The majority of studies above were performed in cell culture and the importance of I3C/DIM regulation of non-coding RNA expression in chemoprevention or therapeutic intervention in human cancer is currently unknown.

### *In vivo* Studies: Preclinical Models

There are excellent reviews on I3C/DIM as chemopreventive agents in preclinical models of cancer (9, 10, 12). In addition to liver, mammary and colon, dietary I3C reduces lung carcinogenesis by the tobacco-specific nitrosamine, 4-(methylnitrosamino)-1-(3-pyridyl)-1-butanone (NNK) and the PAH, benzo[a]pyrene (BaP) (68).

### Cancer Prevention in Rainbow Trout

The rainbow trout cancer model was developed at Oregon State University through pioneering studies by Dr. Russell Sinnhuber and others in the late 1960s-early 1970s (69). Later the model was employed in chemoprevention studies in efforts led by Dr. George S. Bailey (70). There are numerous advantages with this animal model including a low spontaneous background incidence in liver (historically 0.1%), and a high sensitivity to known human carcinogens including aflatoxins, particularly aflatoxin B_1_ (AFB_1_, a known human liver carcinogen), polycyclic aromatic hydrocarbons (PAHs), nitrosamines and direct acting carcinogens such as N-methyl-N'-nitro-nitrosoguanidine (MNNG) (71, 72). Additionally, the low per diem costs for raising trout to 10–12 months of age compared to mice allowed for design of studies employing numbers of animals not achievable in rodent models. Examples include the ED_001_ studies with over 40,000 animals that established the dose-response relationship for dibenzo [*def,p*]chrysene (DBC) to 1 cancer in 5,000 animals and showed a significant deviation from the linear extrapolation model used by regulatory agencies (the calculated dietary dose of DBC resulting in 1 cancer in 10^6^ was 1000-fold higher than the LED_10_ estimate) (73). Interestingly, a subsequent study with dietary AFB_1_ showed a linear dose-response to 1 cancer in 1000 animals although the slope was >1 (1.31) and, again, the dose producing a 1 in 10^6^ cancer estimate was higher (17-fold) than would have been predicted by the LED_10_ (74).

The trout has been used extensively in dietary cancer chemoprevention and promotion studies. Again, the properties of the model could be exploited to address questions that would be difficult in rodent models. When fed prior to and during carcinogen exposure, I3C was an effective chemopreventive agent against a number of carcinogens (70–72). Surprisingly, long-term post-initiation feeding of I3C promoted hepatocarcinogenesis in this model (75–77). Chemoprevention was via the classic “blocking” mechanism resulting from I3C modulation of metabolism. In the case of AFB_1_, induction of trout hepatic CYP1A1 increased detoxication via induced hydroxylation at the 4 position to AFM_1_ and scavenging of the ultimate carcinogenic metabolite, AFB_1_-8,9-*exo*-epoxide (78, 79). I3C administration to rats showed markedly reduced hepatic AFB_1_-DNA adduction associated with induction of both CYP1A1 production of AFM_1_ and GSTa-associated conjugation of AFB_1_-8,9-*exo*-epoxide (80, 81). Subsequent studies documented that DIM promoted hepatocarcinogenesis in trout via functioning as a strong phytoestrogen (82). Utilizing a custom microarray the correlation (*r* = 0.87) between DIM and E_2_ in modulation of hepatic gene expression in trout was highly significant (*p* < 0.0001) (83).

### Cancer Prevention in Rodents

Xenotransplant studies utilize immune-deficient mice, such as the nude or NOD mouse. These mice are implanted with a human cancer cell line and then administered I3C or DIM by diet to examine the inhibition of tumor growth over time (25, 84, 85). Advantages of this model include the use of human cancer cells and ability to easily measure the rate of growth of these surface (often implanted in the flank) tumors. An obvious disadvantage is the absorption, distribution, metabolism and excretion of DIM could exhibit species differences and the implanted tumor cells are growing in a milieu distinct from the actual human cancer. We examined human T-ALL cells *in vitro* for sensitivity to I3C or DIM inhibition of proliferation and viability (85). Concentrations of I3C required to inhibit proliferation or viability of human T-ALL cells (CCRF-CEM cells) were 8-fold higher than DIM (Table 1). Following implantation into SCID (NOD.CB17-*Prkdc*^*scid*^/SzJ) mice the efficacy of dietary DIM and I3C in inhibition of tumor growth was assessed. The tumor volume and doubling time of human CCRF-CEM T-ALL cell xenografts in these mice were both significantly impacted by 100 ppm dietary DIM. Dietary I3C (500 and 2,000 ppm) had a lower inhibitory effect on the growth of the T-ALL xenotransplants (Table 1) (85). The IC_50_ measurements of proliferation and viability were assessed following 24–48 h incubations during which time a significant amount of dimerization of I3C could have accorded.

**Table 1:** I3C and DIM inhibition of human T-ALL cell growth *in vitro* and *in vivo*.

**Indole**	**Proliferation^**a**^ IC_**50**_ (μM)**	**Viability^**a**^ IC_**50**_ (μM)**	**Tumor Volume Reduction (%)^**b**^**	**Tumor Doubling Time Increase (%)**
I3C	122	223	24 (500 ppm) 27 (2,000 ppm)	18 (500 ppm) 31 (2,000 ppm)^c^
DIM	15	27	44 (100 ppm)^c^	59 (100 ppm)^d^
Ratio I3C/DIM	8.1	8.3	–	–

a *Following a 48 h incubation*.

b *Volume determined at conclusion (28 days post engraftment)*.

c *p < 0.01*.

d *p < 0.001*.

As is the case with trout, long-term post-initiation administration of I3C to rats promoted hepatocarcinogenesis (86, 87). In contrast, in the infant mouse model, long-term feeding of I3C significantly (*p* < 0.0005) reduced liver tumors induced by diethylnitrosamine (88). A multi-organ, multi-carcinogen rat model found that prevention vs. promotion was carcinogen- and target tissue-dependent (89). In models of colon cancer, I3C and DIM are chemopreventive in an AHR-dependent fashion which also is responsive to microbial production of indole AHR ligands from dietary tryptophan metabolism (90, 91).

Our laboratory developed a transplacental mouse model of cancer chemoprevention. C57BL/6J (*Ahr*^*b*/*b*^) mice were bred with 129 (*Ahr*^*d*/*d*^) mice and dams dosed with the potent PAH, DBC (30-times more potent that BaP), 2–3 days prior to parturition. Offspring that were exposed *in utero* (and to some degree during lactation) developed an aggressive T-cell acute lymphoblastic leukemia (T-ALL) which began at 3 months of age (92–94). All of the surviving offspring exhibited multiple lung tumors at 10 months of age. The severity of the response was a function of both the maternal and offspring phenotype (i.e., *Ahr*^*b*^ allele is “responsive” and *Ahr*^*d*^ “non-responsive”) (92) and was absent in Cyp1b1 null offspring (93). Administration of phytochemicals known to be chemopreventive in adult models to the maternal diet (gestation day 9 to weaning), including I3C (95, 96), green tea (97) and chlorophyllin (98) provided protection for the offspring from both the young adult onset T-ALL mortality and the mid-life lung cancer. I3C supplementation (2,000 ppm) of the maternal diet (offspring fed control diet throughout lifetime) was the most efficacious and the response was independent of Ahr genotype (95, 96). Neither Brussels sprouts nor broccoli sprouts (10% in AIN93G diet) were protective when added to maternal diet and the same was observed for sulforaphane (96). The lack of response with the whole food is likely related to the small amount of I3C achievable in such diets (e.g., it would require consumption of an estimated 10–60 Kg daily of Brussels sprouts to achieve an I3C level of 1,000 ppm in the diet) (96). Administration of dietary I3C to pregnant rats up-regulated CYP1A1 and CYP1B1 expression in neonatal liver (99). In examination of chemoprevention of breast cancer in rats induced by 7,12-dimethylbenzanthracene (DMBA) or the direct-acting carcinogen, N-methylnitrosourea (MNU), I3C was effective whereas DIM was not (100). These results caution against attributing the chemoprevention of I3C in every model to DIM formation. Protection against DBC-dependent T-ALL in female, but not male, offspring born to mothers fed I3C during gestation required expression of ERβ (101). Such results would be consistent with I3C and DIM ER-dependent chemoprevention in breast (7, 8, 37–44).

### Cancer Prevention in Clinical Trials

Epidemiological studies show an inverse correlation between cruciferous vegetable intake and some cancers (1, 12, 14, 15, 102). Both I3C and DIM have been studied in human clinical trials, primarily to test efficacy against breast and prostate cancer (Table 2) (8, 11, 48, 52, 53, 108–110). The focus on breast cancer in women derives from the demonstrated capacity for I3C or DIM to CYP1-dependent estrogen metabolism. 2-Hydroxyestrogen exhibits reduced pharmacological activity whereas 16α-hydroxyestrogen retains estrogenic activity. The ratio of 2-hydroxy/16α-hydroxy estrogen has become a biomarker for risk of estrogen-dependent cancer and cervical intraepithelial neoplasia (CIN). Clinical trials with both I3C and DIM result in an increase in this ratio and, in the case of CIN, demonstrably improved clinical outcomes. There is little evidence to suggest that supplementation for months represents a significant risk from women. One study (48) in Table 2 highlights a potential concern regarding DIM alteration in metabolism of co-administered pharmaceutics (tamoxifen) not surprising given that DIM inhibits, as well as induces, a number of CYPs. DIM inhibits human CYP3A4, responsible for metabolism of 60% of prescribed drugs, with an IC_50_ of 14.5 μM (Table 3). The concern regarding DIM and adverse drug responses is discussed further in the section titled Potential Risks of Long-Term I3C/DIM Supplementation: Inhibition of CYP Activity and Levels of Flavin-Containing Monooxygenase: A Potential ‘Drug-Drug' Interaction? Studies to date on DIM supplementation in treatment of prostate cancer progression also suggests the potential for some benefit in slowing progression. Following DIM supplementation in men scheduled for prostatectomy, not only were androgen receptor levels in prostate reduced but there was exclusion of the receptor from the nucleus (109) (Table 2). With one exception, the clinical trials in Table 2 examine the cancer therapeutic potential of DIM and not strictly chemoprevention. Double-blind, placebo-controlled studies in disease-free subjects are needed to better determine the potency and efficacy of DIM as a chemopreventive supplement. One important finding from these studies, primarily in humans with existing disease, is that supplementation with DIM at 200–400 mg/day is not likely to represent a risk (again, with the caveat that co-administration of some drugs could cause an adverse effect).

**Table 2:** Clinical trials with indole-3-carbinol and 3,3'-diindolylmethane.

**Subjects (*n*)**	**Dailey dose**	**Duration (days)**	**Endpoint**	**Adverse effects**	**References**
Women (5)^a^	I3C 200 or 400 mg bid	28	**↑** E2 2-OH/16α-OH and **↓**CIN	None Reported	(103, 104)
Women (17)^b^	I3C 200 then 400 mg bid	28 at 200 mg then 28 at 400 mg	**↑** E2 2-OH/16α-OH **↑** CYP1A2	No adverse effects compared to placebo	(105)
Women (14)^c^	I3C 400 mg bid	14	Pharmacokinetics	None reported	(106)
Women (10)^d^	DIM 108 mg	30	**↑**E2 2-OH	None reported	(52)
Women (47)^e^	BR-DIM^f^ 150 mg bid	Up to 18 months	**↑** E2 2-OH/16α-OH and **↓** TAM metabolites	No adverse effects compared to placebo	(48)
Women (40)^g^	100 and 200 mg	180	**↓**CIN	No serious adverse events compared to placebo	(107)
Men (12)^h^	BR-DIM Escalating dose 75–300 mg bid	Median 120; range of 1–18.5 months	Pharmacokinetics; MTD; RP2D, QoL, PSA^i^	None at 225 mg; 2/4 men mild adverse effect at 300 mg	(108)
Men (26)^j^	BR-DIM 225 mg bid	Median 19; range 4–104	Pharmacokinetics; AR IHC^k^ PSA	Minimal (2 treatment-related reports of headache)	(109)

a *Women with varying degrees of cervical dysplasia*.

b *Healthy non-smoking women, ages 20–58 years of age, with enhanced breast cancer risk*.

c *Healthy non-smoking women, ages 18–65 years of age, with enhanced breast cancer risk*.

d *Postmenopausal women, ages 50–70 with a history of early-stage breast cancer*.

e *Cancer-free women, average age 53 with average BMI of 26, taking tamoxifen for prevention of breast cancer*.

f *BR-DIM, BioResponse-DIM® from BioResponse, L.L.C., is a commercially available formulation with clinically demonstrated enhanced bioavailability*.

g *Women, ages 18–39 years, with histologically verified cervical intraepithelial neoplasia (CIN) type I or II*.

h *Men, age 62–91, with castrate-resistant, non-metastatic, PSA relapse prostate cancer*.

i *Daily Maximum Tolerated Dose (MTD) was 300 mg and the Recommended Phase II Dose (RP2D) was 225 mg. The rate of Prostate Specific Antigen (PSA) rise in patients taking 225 mg daily initially declined but eventually progressed to placebo rates of increase and/or presented with metastatic disease*.

j *Men, age 50–73, had histologically or cytologically confirmed, treatment-naïve, T1 or T2 prostate cancer and were scheduled for prostatectomy*.

k *Median Androgen Receptor (AR) protein levels declined 15% with treatment and almost all AR was excluded from the nucleus; PSA levels declined modestly (median of 5.9 ng/mL compared to 6.4 ng/mL prior to BR-DIM administration)*.

*“CervikonDIM” (CJSC “IlmixGroup,” Russia) in the form of a vaginal suppository*.

**Table 3:** Inhibitory constants for DIM with trout, rat and human CYPS.

**Species**	**Enzyme source (Inducer)**	**Activity (CYP selectivity)**	**Inhibition constant (μM)**	**Type of inhibition**	**References**
Trout	Liver microsomes (BNF)^a^	EROD^k^ (CYP1A1)	K_is_= 2.7 ± 0.5 K_ii_ = 14 ± 2.2	Non-competitive	(111)
Rat	Liver microsomes (BNF)^b^	EROD (CYP1A1)	K_i_ = 2.2 ± 0.2	Competitive	(111)
Rat	Liver microsomes (PB)^c^	PROD^l^ (CYP2B1)	K_is_ = 0.62 ± 0.08 K_ii_ = 1.2 ± 0.60	Non-competitive	(111)
Rat	Liver microsomes (BNF)^d^	EROD (CYP1A1)	K_i_ = 1.60 ± 0.31 K_iu_ = 22.0 ± 6.2	Competitive Uncompetitive	(112)
Rat	Liver microsomes (PB)^e^	PROD (CYP2B1)	K_i_ = 0.36 ± 0.09 K_iu_ = 0.60 ± 0.12	Competitive Uncompetitive	(112)
Rat	Liver microsomes (PB)^e^	BROD^m^ (CYP3A4)	K_iu_ = 0.47 ± 0.02	Uncompetitive	(112)
Rat	Liver microsomes (BNF)^b^	AFB_1_-8,9-E (CYP1A2 & 3A4)^n^	K_is_= 137 ± 43 K_ii_ = 58 ± 11	Non-competitive	(111)
Rat	Liver microsomes (BNF)^b^	AFM_1_ (CYP1A)^o^	K_i_ = 128 ± 24	Competitive	(111)
Human	Expressed 1A1^f^	EROD (CYP1A1)	K_is_ = 7.4 ± 2.0 K_ii_ = 13 ± 2.7	Non-competitive	143
Human	Expressed 1A2^g^	Acetanilide-4-OH (CYP1A2)	K_i_ = 7.6 ± 4.1	Competitive	(113)
Human	Expressed 1A1^h^	EROD (CYP1A1)	IC_50_ = 21.4	–	(113)
Human	Expressed 1A2^i^	MROD^p^ (CYP1A2)	IC_50_ = 40.9	–	(113)
Human	Expressed 3A4^j^	BROD (CYP3A4)	IC_50_ = 14.5	–	(113)

a *Adult (200-300 g) rainbow trout fed 700 ppm β-naphthoflavone (BNF) for 7 days*.

b *Male Fischer 344 rats injected ip daily for 4 days with 40 mg/kg BNF in corn oil*.

c *Male Fischer 344 rats administered 0.1% phenobarbital (PB) for 7 days in drinking water*.

d *Female Sprague-Dawley rats gavaged with 40 mg/kg BNF daily for 4 days*.

e *Female Sprague-Dawley rats injected ip with 75 mg/kg PB daily for 4 days*.

f *Microsomes from a human lymphoblastoid cell line (Gentest (Woburn, MA) with expressed recombinant human CYP1A1*.

g *Microsomes from a human lymphoblastoid cell line (Gentest (Woburn, MA) with expressed recombinant human CYP1A2*.

h *Microsomes from yeast (Gentest (Woburn, MA) expressing recombinant humans CYP1A1*.

i *Microsomes from yeast (Gentest (Woburn, MA) expressing recombinant humans CYP1A2*.

j *Supersomes (Gentest (Woburn, MA) expressing recombinant humans CYP3A4*.

k *Ethoxyresorufin-O-deethylase*.

l *Pentoxyresorufin-O-depentylase*.

m *Benzyloxy-O-debenzylase*.

n *Aflatoxin B_1_-epoxygenation with glutathione trapping*.

o *Aflatoxin B_1_-4-hydroxylation*.

p *Methyoxyresorufin-O-demethylase*.

### Pharmacokinetics of DIM in Rodents and Humans

**Figure 2:**
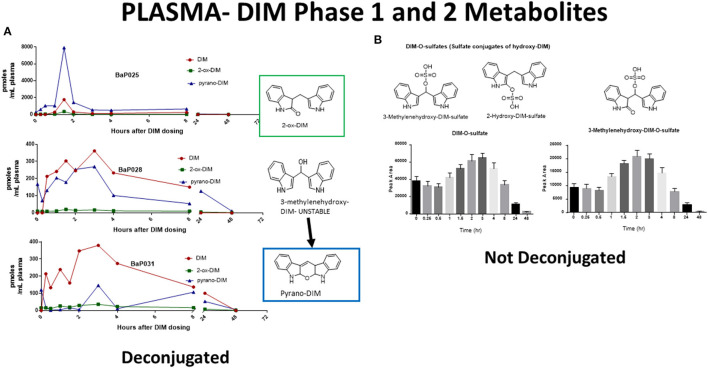
DIM Metabolites in Plasma Over 48 h. **(A)** Levels of DIM and mono-hydroxylated metabolites following deconjugation. Shown are individuals with extensive (BaP025), intermediate (BaP028) and slow (BaP031) metabolism. 3-Methylenehydroxy-DIM is unstable and is quantitated as pyrano-DIM. **(B)** Mean and S.D. of sulfate conjugates in plasma over 48 hr. Glucuronides were also present but are not shown. See Maier et al., (20) for complete details.

**Figure 3:**
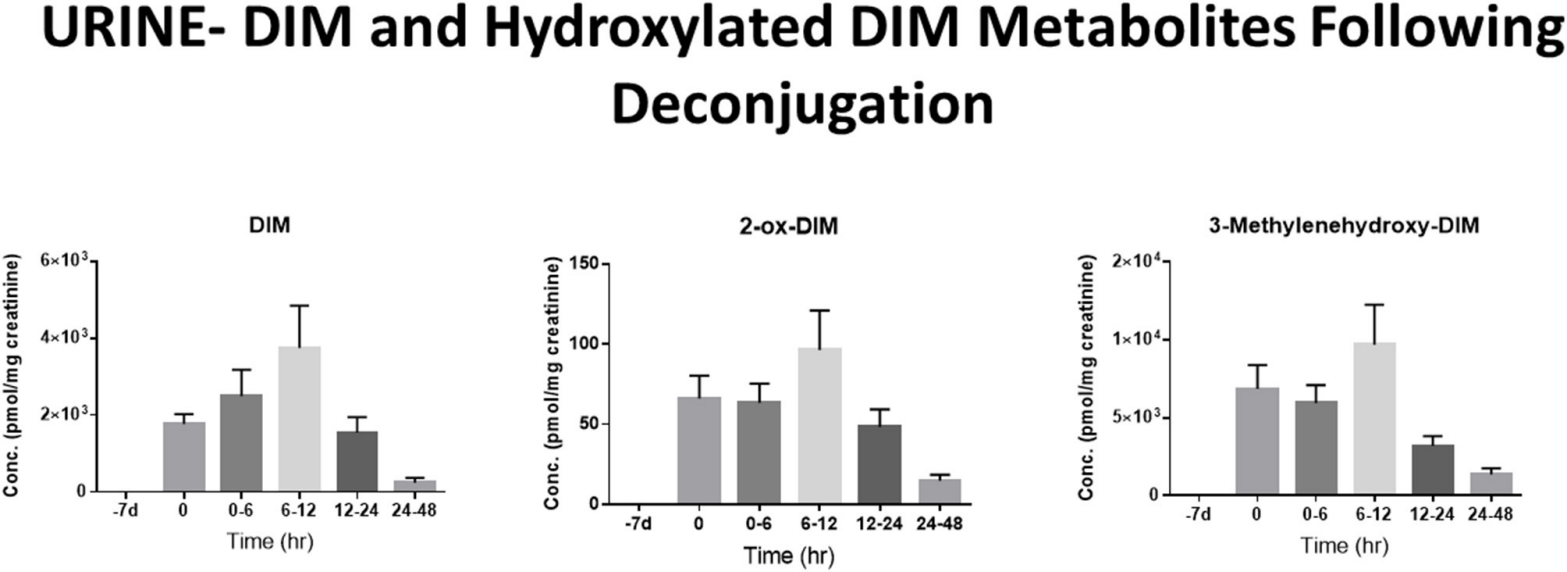
DIM Metabolites in Urine Over 48 h. Levels of DIM and mono-hydroxylated metabolites following deconjugation with β-glucuronidase and sulfatase. DIM and 2-ox-DIM were quantitated with standard curves and are reported in pmol/mg creatinine. 3-Methylenehydroxy-DIM is unstable and is quantitated as pyrano-DIM assuming instrument response similar to 2-ox-DIM. See Maier et al., (20) for complete details.

The pharmacological mechanism(s) of action of DIM (and to a large extent I3C) has been attributed to DIM and the impact of the pharmacodynamics of I3C/DIM has largely been ignored. As discussed above, DIM is the major, if not sole, I3C product detected *in vivo* after oral administration and urinary levels of DIM have been proposed as a biomarker for glucobrassicin consumption (114, 115). Pharmacokinetics of I3C following administration to humans has been done by analysis of plasma and urinary DIM levels (103, 107). Incubations of DIM with liver microsomes from female rats fed diets containing β-naphthoflavone (BNF), phenobarbital (PB) or I3C, produced two unidentified mono-hydroxylated metabolites (112). Human MCF-7 breast cancer cells incubated with 1 μM [^3^H]-DIM for 24–72 h yielding three mono-hydroxylated CYP products, 3-[(1*H*-indole-3-yl) methyl]indolin-2-one (2-oxo-DIM, a tautomer with 2-hydroxy-DIM), bis (1*H*-indol-3-yl)methanol (3-methylenehydroxy-DIM) and 3-hydroxy-DIM along with two di-hydroxylated metabolites (3-methylenehydroxy-2-ox-DIM and 3-hydroxy-2-ox-DIM) and their sulfate conjugates (43). Co-incubation with isoform-selective CYP inhibitors suggested that CYP1A2 played the primary role in metabolism of DIM in MCF-7 cells. Surprisingly, *in vivo* pharmacokinetic studies in both mice, rats and humans have failed to report the formation of any phase 1 or phase 2 metabolite in plasma or urine (52, 103, 106, 108, 109, 116–119). In a recently published study with humans taking BioResponse DIM®, a formulation with clinically demonstrated enhanced absorption, we found extensive and rapid appearance of metabolites in both plasma (Figure 2) and urine (Figure 3) (20). The profile of these metabolites was very similar to what Staub et al., (43) observed *in vitro* with MCF-7 cells (43). Unlike their study, the major site of hydroxylation in our *in vivo* study was not the 2-position but rather the 3-methylene position of DIM. Both of these mono-hydroxylated DIM metabolites were rapidly conjugated and sulfation seem to predominant (as in MCF-7 cells) although glucuronides were also present (Figure 2) (20). No di-sulfate, di-glucuronide or sulfate-glucuronide conjugates were found in plasma or in urine. As with the *in vitro* study with MCF-7 cells, we found free and conjugated di-hydroxylated-DIM. We did not report finding 3-hydroxy-DIM either free or conjugated. Interestingly, 3-methylenehydroxy-DIM was not stable and spontaneously rearranged to a putative pyrano metabolite, 5a,6a,11b-tetrahydro-5-H-pyrano [,2,3-b:6,5-b'] diindole (pyrano-DIM). As has been observed in previous pharmacokinetic studies with I3C or DIM in humans (52, 103, 106, 108, 109, 116–119), there was significant inter-individual variability (Figure 2).

In MCF-7 cells the major mono-hydroxylated metabolite of DIM, 2-ox-DIM, failed to demonstrate any estrogenic activity. When we examined this same metabolite in an AHR reporter system we found it to be a more potent agonist than parent DIM and as potent or more so than known indole AHR agonists derived from microbial or host metabolism of tryptophan (i.e., indole-3-acetonitrile, kynurenine, indole, indole-3-aldehyde and indole-3-acetate) (20, 120). There is a possibility that metabolites of DIM may contribute to its beneficial chemopreventive properties. The extensive metabolism of DIM also highlights the potential for DIM-drug adverse interactions via competitive inhibition with CYPs, SULTs or UGTs. The high inter-individual variability in pharmacokinetics may reflect differences in genetic- and environmental-dependent levels of the CYPs, SULTs and UGT isoforms important in DIM metabolism.

### Additional Health Benefits: I3C and DIM in Prevention or Therapy of Disease other than Cancer

I3C and DIM impact many cellular pathways [cell cycle, epithelial mesenchymal transition, cell proliferation, apoptosis, autophagy, oxidative stress, inflammation, metastasis (migration, adhesion and invasion), angiogenesis, multiple drug resistance, endoplasmic reticulum stress, and DNA repair]. (10, 16–18, 28, 29, 31, 32). Thus, it is not surprising to find proposed applications in the treatment or prevention of other toxicities, diseases or cancers (other than breast, ovarian, prostate, lung, liver and colon) including neurotoxicity, ionizing radiation, thyroid disease, endometriosis, Epstein-Barr viral Burkitt's lymphoma, papilloma viral-dependent cancers, cervical dysplasia/cancer and metabolic syndrome (107, 121–128). Descriptions of clinical trials for DIM and systemic lupus (129), childhood laryngeal papilloma (130), thyroid disease (131) and recurrent respiratory papillomatosis (132) can be found at www.ClinicalTrials.gov.

In recent years indoles, other than I3C and DIM, derived from microbial (e.g., indole, indole-3-acetate, indole-3-aldehyde, tryptamine) or host (e.g., kynurenine, kynurenic acid, xanthurenic) metabolism of dietary tryptophan were shown to be AHR ligands (120, 133). AHR activation by these indoles in the intestine protects against inflammatory-related chronic disease such as Crohn's disease, irritable bowel syndrome, metabolic syndrome and obesity (134–137). Absence of intestinal AHR activity results in dysbiosis of the gut microbiome and a compromised epithelial barrier with increased intestinal inflammation, enhanced “leakage” with susceptibility to pathogenic bacteria (e.g., *Citrobacter rodentium*) and colon cancer (138). Addition of dietary AHR ligands such as DIM ameliorate dysbiosis, inflammation, compromised barrier function and colon cancer from deprivation of bacteria-derived indoles (90, 91). Cruciferous vegetables or DIM intake alters the gut microbiome and the relationship is truly bidirectional (139, 140). I3C ameliorated murine colitis and DIM reversed dysbiosis in a murine model of colorectal cancer (91). These observations provide evidence for I3C/DIM promotion of intestinal health.

### Potential Benefits of Combination With Whole Food and/or Other Phytochemicals: Example of Synergism With Sulforaphane

In a comprehensive review in this issue, Bouranis et al., detail the formation of sulforaphane (SFN) from the glucosinolate, glucoraphanin, also present in crucifers. As is the case with I3C and DIM, SFN has been demonstrated to be an effective cancer chemopreventive agent in preclinical models and is the focus of a number of clinical trials. I3C and DIM have been shown to have the potential to act synergistically with SFN in Nrf-2 signaling in a human cancer cell line (HepG2-C8) (141) and inhibition of cell proliferation in human colon cancer cells (40–16) (30). These observations open up new strategies for the design of human clinical trials and also cautions against reliance solely on a reductionist approach (a single phytochemical) when studying the human health benefit of foods.

### Potential Risks of Long-term I3C/DIM Supplementation: Inhibition of CYP Activity and Levels of Flavin-containing Monooxygenase: A Potential “Drug-Drug” Interaction?

As discussed above I3C and DIM function as AHR agonists and induce CYPs in the 1 family as well as phase 2 conjugating enzymes [glutathione-S-transferases (GSTs), UDP-glucuronosyltransferases (UGTs) and sulfotransferase (SULTs)], a major contributor to the “blocking” mechanism of chemoprevention. I3C/DIM activation of Nrf-2-dependent signaling, either directly or via AHR upregulation of Nrf-2, induces a number of phase 2 enzymes that contribute to blocking by enhancing detoxication and excretion through conjugation. In addition to induction of CYPs, DIM has been demonstrated *in vitro* to be an effective inhibitor of the catalytic activity of a number of CYPs with K_i_s in the low μM range (Table 3) (111, 112). The contribution of DIM-dependent inhibition of carcinogen metabolism in cancer chemoprevention is not known. However, this observation could raise potential concerns about potential adverse drug interactions with long-term use.

CYPs are not the only phase 1 enzymes impacted by I3C/DIM. The flavin-containing monooxygenase, like CYP, is a superfamily of monooxygenases present in the endoplasmic reticulum of tissues such as liver, lung, intestine and kidney, and utilizes the reducing power of NADPH to insert one atom of O_2_ into a substrate and the other into formation of H_2_O (142). Each family is comprised of a single enzyme and in humans there are 5 expressed FMOs (FMO1, FMO2, FMO3, FMO4 and FMO5) (143). In mammals, with the exception of primates, FMO1 is the major FMO in liver and metabolizes a wide range of xenobiotics (143). Humans express FMO1 in liver prior to parturition after which time it is replaced with FMO3 (144). FMO1 in rat (but not guinea pig, mouse or rabbit) and FMO3 in humans are inhibited by dietary I3C/DIM (145–149). Human FMO3 is responsible for N-oxygenation of the noxious odorant trimethylamine (TMA) to the odorless trimethylamine-N-oxide (TMAO) (150). Genetic variants of human FMO3, resulting in reduced conversion of TMA to TMAO, are associated with the genetic disease trimethylaminuria and individuals with the disease exhibit severe body odor problems (as well as associated psycho-social issues) due to elevated levels of TMA in urine and sweat (150). Feeding Brussels sprouts to humans resulted in a marked *in vivo* increase in the ratio of TMA/TMAO and the mechanism was shown *in vitro* to be I3C, DIM and LT inhibition of FMO3 catalytic activity (149). Thus, trimethylaminuria patients could see worsening symptoms if ingesting significant amounts of Brussels sprouts or taking I3C or DIM supplements. On the contrary, evidence has accumulated that FMO3-dependent formation of TMAO is associated with increased risk of cardiovascular disease (151) in which case individuals at risk (which greatly outnumber trimethylaminuria patients) could benefit from diets high in crucifers or I3C/DIM supplements. This may be the rationale behind a clinical trial, “Targeting FMO-Mediated TMAO Formation in Kidney Disease (TMAO) Study (TMAO)” (NCT03152097) (152). Induction of CYPs, with concurrent down-regulation of FMO by dietary indoles, may lead to alterations in the profile of metabolites from drugs/xenobiotics that are substrates for both monooxygenases and could represent an adverse “drug-drug” interaction (Table 4) (145). N,N-dimethylaniline (DMA), (*S*)-nicotine (NIC) and tamoxifen (TAM) are all tertiary amines which tend to be N-dealkylated by CYPs whereas FMOs catalyze formation of the N-oxide. Feeding I3C or DIM to rats for 4 weeks produced a dose-dependent reduction in liver microsomal FMO1 protein (Table 4). The ratio of FMO/CYP-mediated metabolism of DMA was reduced in a dose-dependent fashion by dietary I3C from 1.1 to 0.22 and 0.07 at the low and high dose, respectively. DIM lowered the ratio of FMO/CYP DMA metabolism to 0.14 and 0.02 at the low and high dose, respectively. We and others had previously documented the role of FMO in metabolism of NIC (154, 155). Again, the FMO product is the N-oxide whereas, CYP N-demethylates or produces the Δ (1, 5)-iminium ion of NIC. The rate of CYP metabolism of NIC did not change with diet but N-oxygenation was markedly inhibited and no N-oxide could be detected with liver microsomes from rats fed 2,500 ppm I3C or 1,000 ppm DIM (Table 4). TAM (an ER antagonist) is used in the chemoprevention or treatment of ER-dependent breast cancer but use is limited by ovarian toxicity, primarily due to 4-hydroxylation (156). As with NIC, formation of TAM-N-oxide is regarded as detoxication and inhibition of the metabolism to the N-oxide is likely to enhance toxicity of both NIC and TAM (157).

**Table 4:** Dietary I3C and DIM alteration of FMO1 in rat liver microsomes and FMO/CYP-mediated metabolism of N,N-dimethylaniline, nicotine and tamoxifen (145, 153).

**Diet^**a**^**	**FMO1 Protein** ** (% Control)^**b**^**	**UL-ring ^**14**^C-N,N-Dimethylaniline** ** (FMO/CYP)^**c**^**	**(*S*)-5-^**3**^H-Nicotine** ** (FMO/CYP)^**d**^**	**^**3**^H-N-methyl-Tamoxifen** ** (FMO/CYP)^**e**^**
Control	100	1.11	1.43	0.79
1,000 ppm I3C	93	0.22	1.11	0.53
2,500 ppm I3C	10	0.07	0	0.20
1,000 ppm DIM	13	0.14	0	0.32
2,500 ppm DIM	3	0.02	0.08	0.31

a *Male Fischer 344 rats were fed AIN-76A diet containing I3C or DIM for 4 weeks*.

b *FMO1 protein levels in liver microsomes measured by western blotting using polyclonal antibody to pig FMO1*.

c *[^14^C]-Dimethylaniline-N-oxide (FMO) and methylaniline (CYP) measured by HPLC with on-line radiochemical detection*.

d *(S)-[^3^H]-Nicotine-N-1'-oxide (FMO), nornicotine (CYP) and nicotine-Δ^1,5^-iminium ion (CYP) measured by HPLC with on-line radiochemical detection*.

e *[^3^H]-Tamoxifen-N-oxide (FMO), N-desmethyl-tamoxifen (CYP) and 4-hydroxy-tamoxifen (CYP) were determined by TLC and radioscanning using a System 2,000 imaging scanner (Bioscan, Inc., Washington, DC)*.

## Conclusions

I3C and DIM, the major indoles released upon hydrolysis and ingestion of glucobrassicin from Brussels sprouts and other cruciferous vegetables, have been studied extensively and their beneficial impact on cancer (and other diseases) is well-documented. The doses employed in preclinical and clinical models are not realistically achievable by ingestion of the whole food so supplementation is necessary to achieve the benefits attributed to I3C and DIM. Ingestion of I3C results in 20–40% conversion to DIM but dozens of other acid condensation products are also produced, the pharmacological properties of which are largely unknown, so at present supplementation with DIM would seem preferable. DIM is chemopreventive against cancer via a variety of mechanisms. AHR, ER and AR binding (with resultant agonism or antagonism) are associated with mechanisms ascribed to DIM. There are however unresolved questions related to the use of DIM in prevention or treatment of disease. DIM has been shown to act in both AHR-dependent up-regulation of drug/carcinogen metabolizing enzymes (e.g., CYPs, GSTs, UGTs, SULTs) and *in vitro* inhibition of catalytic activity (CYPs). What is the net effect *in vivo*? Does DIM supplementation pose any concerns for an adverse “drug-drug” type interaction and, if so, who would be at risk? Based on results from clinical trials, long-term supplementation with 300 mg daily would not produce adverse effects. One caveat is that these trials typically employed subjects with disease or with exclusion criteria that would prevent elucidation of adverse drug interactions. Another variable of potential concern is the bidirectional interaction between DIM and an individual's microbiome. The DIM-intestinal AHR-microbiome axis is an important component for future development of a “personalized nutraceutical” (concept similar to personalized medicine) approach to achieving optimal health.

It is recognized that other components in crucifers, such as sulforaphane derived from hydrolysis of glucoraphanin, contribute to the health benefits from crucifers and these vegetables should be an important component of the diet. Future research efforts should focus on the potential for inhibition, additivity or synergy from combinations of whole foods or other phytochemical nutraceuticals given with I3C/DIM in cancer chemoprevention.

Many of the mechanisms of action attributed to I3C and DIM have been elucidated from *in vitro* cell culture studies. Based on pharmacokinetic data with I3C/DIM following oral ingestion, and recent results from our laboratory on extensive “first-pass” metabolism in humans, the relevance of results obtained following prolonged incubation with any level of I3C or non-physiologically relevant concentrations of DIM (≥ 15 μM) is questionable.

## Author Contributions

The author confirms being the sole contributor of this work and has approved it for publication.

## Funding

This work was supported by NIH grant ESR01ES028600 and NIFA project W4122.

## Conflict of Interest

The author declares that the research was conducted in the absence of any commercial or financial relationships that could be construed as a potential conflict of interest.

## Publisher's Note

All claims expressed in this article are solely those of the authors and do not necessarily represent those of their affiliated organizations, or those of the publisher, the editors and the reviewers. Any product that may be evaluated in this article, or claim that may be made by its manufacturer, is not guaranteed or endorsed by the publisher.
